# Medical Remembrancer, or Book of Emergencies

**Published:** 1845-12-01

**Authors:** 


*Medical Remembrancer, or Book of Emergencies; in which are concisely pointed out the immediate remedies to be adopted in the first moments of danger—from poisoning, drowning, apoplexy, burns, and other accidents: with the tests for the principal poisons, and other useful information; by Edward B. L. Shaw, M. R. C. S. &c. revised and improved by an American Physician; Published by Samuel S. and William Wood, New-York,* 1845.

The above title page is sufficiently comprehensive to denote the purpose and scope of the work. The author intimates in his preface that it is to be carried in the pocketf and consulted as emergencies arise. We do not coincide with him in the propriety o, this; but we think the practitioner will find it useful to have a work of this description lying upon his office table, with which occasionally to refresh his recollection of those matters which he may at any moment wish to call into requisition, without much time for premeditation. In this way, this little volume will fulfil an useful purpose in so far as it goes. Its utility would be greatly enhanced, if it were somewhat more extended.

